# A new method for in vitro feeding of *Rhipicephalus australis* (formerly *Rhipicephalus microplus*) larvae: a valuable tool for tick vaccine development

**DOI:** 10.1186/s13071-017-2081-0

**Published:** 2017-03-23

**Authors:** Jos J. A. Trentelman, Jos A. G. M. Kleuskens, Jos van de Crommert, Theo P. M. Schetters

**Affiliations:** 1 Center for Experimental and Molecular Medicine, Academic Medical Center Amsterdam, meibergdreef 9, 1105 AZ Amsterdam, Netherlands; 2 Aduro Biotech Europe, Kloosterstraat 9, 1101 RX Boxmeer, Netherlands; 3 ProtActivity R&D, Sering 36, 5432 DD Cuijk, Netherlands; 4 ClinVet International, Uitzich Road, Bainsvlei, 9338 Bloemfontein, South Africa

**Keywords:** Artificial tick feeding, In vitro screening, *Rhipicephalus australis*, *Rhipicephalus microplus*, Larvae

## Abstract

**Background:**

*Rhipicephalus microplus* is a hard tick that has a major impact on cattle health in tropical and subtropical regions because it feeds on cattle and is implicated in the transmission of pathogens that cause diseases such as bovine anaplasmosis and babesiosis. Presently, acaricides are used to control tick infestation but this is becoming increasingly less effective due to the emergence of tick strains that are resistant to one or more classes of acaricides. Anti-tick vaccines are a promising alternative to control tick infestation in cattle. The life-cycle and host preference of *R. microplus*, however, makes vaccine research in cattle costly and would therefore greatly benefit from an in vitro screening system.

**Methods:**

To this aim, a stacked 24-well in vitro feeding system was designed in which the blood meal was administered in a chamber on top of the compartment containing the ticks, exploiting their anti-gravitational tendency. Both compartments were separated by a special feeding membrane, which was made by applying a silicone mixture to a gold beater’s skin (baudruche membrane) with a paint roller to create a slightly uneven surface of 17–40 μm variable thickness. To further stimulate feeding, the membrane was treated with bovine hair extract and the unit was placed at 37 °C with 90% RH and 5% CO_2_.

**Results:**

Using this set-up with *Rhipicephalus australis* (formerly *Rhipicephalus microplus*), a larval engorgement rate of up to 71% could be achieved. The larvae could successfully feed on blood, but also on serum. The latter allows easy screening of the effect of sera that are raised against tick proteins on feeding. As an example, serum from cattle that were vaccinated with the Bm86 midgut protein of *R. microplus* significantly reduced larval engorgement rates by 42%.

**Conclusion:**

The in vitro feeding system’s high throughput design and its ability to measure statistically significant anti-tick effects in sera from immunized cattle enables screening of multiple vaccine candidates in a cost-effective manner.

## Background

*Rhipicephalus microplus* is a hard tick that has a major impact on cattle health in tropical and subtropical regions. This tick species can transmit a range of diseases, including bovine anaplasmosis and babesiosis. Moreover, ticks can also adversely affect cattle production directly by feeding alone [1]. It is therefore of great importance to control tick infestations to ensure livestock health and productivity. To date, tick control heavily depends on the use of tick-resistant breeds and treatment of susceptible breeds with acaricides, but tick resistance to these acaricides is becoming problematic [2].

Anti-tick vaccines are potential alternatives for acaricides to control tick infestation. Anti-tick immunity is known to consist of both cellular and humoral factors [3–6]. Transfer of tick immune serum to naïve animals resulted in tick rejection indicating an important role for antibodies [7, 8]. Early studies have shown that vaccination with crude tick antigen preparations induced antibodies that interfered with feeding and subsequent further development [9, 10]. With the advent of recombinant protein techniques, single protein antigens could be evaluated for protective activity. This led to the discovery of Bm86, a tick midgut antigen first described in 1989 [11]. This antigen is the basis of two commercial anti-tick vaccines (Gavac™, Heber biotec™; TickGard, Merck Animal Health) [12, 13]. In experimental studies, Bm86 vaccination reduces tick numbers up to 74% and reduces tick fertility, combining the overall efficacy of up to 91% depending on the *R. microplus* challenge strain [14]. Protection by Bm86 vaccination is IgG mediated and protection can be correlated to IgG titers [15, 16]. Although Bm86 vaccination reduces the number of acaracide treatments in the field, a lack of a knock-down effect hampers its wide spread use [12, 17, 18]. More research is needed to improve anti-tick vaccines by additional and/or more efficacious antigens.

Currently, vaccination-challenge trials in cattle are being used to evaluate and select *R. microplus* vaccine candidate antigens. Aside from the fact that this restrains the number of experiments that can be done from an animal welfare point of view, it is also very costly and time consuming. As antibodies are an essential mediator in induced and natural anti-tick immunity, an in vitro feeding model of *R. microplus* is an attractive alternative to evaluate the inhibitory effect of such sera on engorgement and further tick development. Different techniques have been tried with varying degrees of success. These techniques can be divided in two approaches: tube feeding and membrane feeding. In vitro feeding of blood with tubes has proven to be a successful method for feeding *R. microplus* in vitro; ticks are forced to feed by placing a glass tube with blood over the hypostome or the entire mouthparts [19–21]. A major drawback of tube feeding is that only semi-engorged adult females can be used; they are eager to imbibe blood and they have the larger feeding apparatus needed for this technique. Larvae and nymphs cannot be fed using capillaries.

Membrane feeding, which more closely mimics the natural feeding process, has been successfully developed for several tick species [22–25]. This technique is not limited to adult ticks, but can also be used to feed larval hard ticks in vitro; either with a completely artificial feeding membrane [23, 24] or derived from animal skin [25, 26]. These studies also showed that the blood meal could be presented below or above the tick compartment. However, in the few described cases of membrane feeding of *R. microplus* larvae, embryonated hen egg membranes or bovine skin slices were used and the ticks were placed on top of the blood meal [27–29]. For adult *R. microplus* ticks, these membranes were subsequently replaced by artificial feeding membranes prepared from gut intestines (baudruche membrane) [30]. The baudruche membrane was coated with layers of contact cement and treated with bovine skin extract to stimulate the feeding process. These attempts met with limited success, and presently there is still no recorded in vitro membrane feeding system that allows reproducible feeding of *R. microplus* larvae*.* Only recently ribosomal DNA and morphological analysis resulted in the reinstatement of *R. australis* as a separate species from *R. microplus* [31]. Morphologically there are only small differences between *R. microplus* and *R. australis* larvae, the hypostome length is however identical, making *R. australis* an excellent model for both species. Here we describe the development of an in vitro membrane feeding system for *R. australis* larvae that can be used to evaluate the inhibitory activity of antisera against tick antigens. The system described here enables high-throughput screening for tick vaccine candidate antigens.

## Methods

### Tick larvae

Tick larvae were obtained from a colony of *R. australis* (formerly *R. microplus*) originating in Australia that was routinely passaged on Holstein calves (Merck Animal Health Innovation GmbH, Schwabenheim, Germany). Fully engorged female ticks were collected from the calves and allowed to oviposit in petri dishes. The resulting egg masses were collected in laboratory tubes and allowed to hatch at 22 °C and 90% humidity. Four to 6-week-old *R. australis* larvae were used in the feeding experiments.

### Preparation of cow hair extract

Hair was collected from the flank of tick naïve Friesian Holstein cattle. Fifty gram of hair was incubated for 20 min at room temperature with 250 ml dichloromethane (Merck, Darmstadt, Germany), supernatant was collected. This was repeated twice with 100 ml dichloromethane. The collected dichloromethane extracts were pooled and centrifuged at 3000× *rpm* for 20 min. Supernatant was collected and concentrated to a low volatile lipid content of 7 mg/ml by heating at 40 °C.

### Feeding unit design

**Fig. 1:**
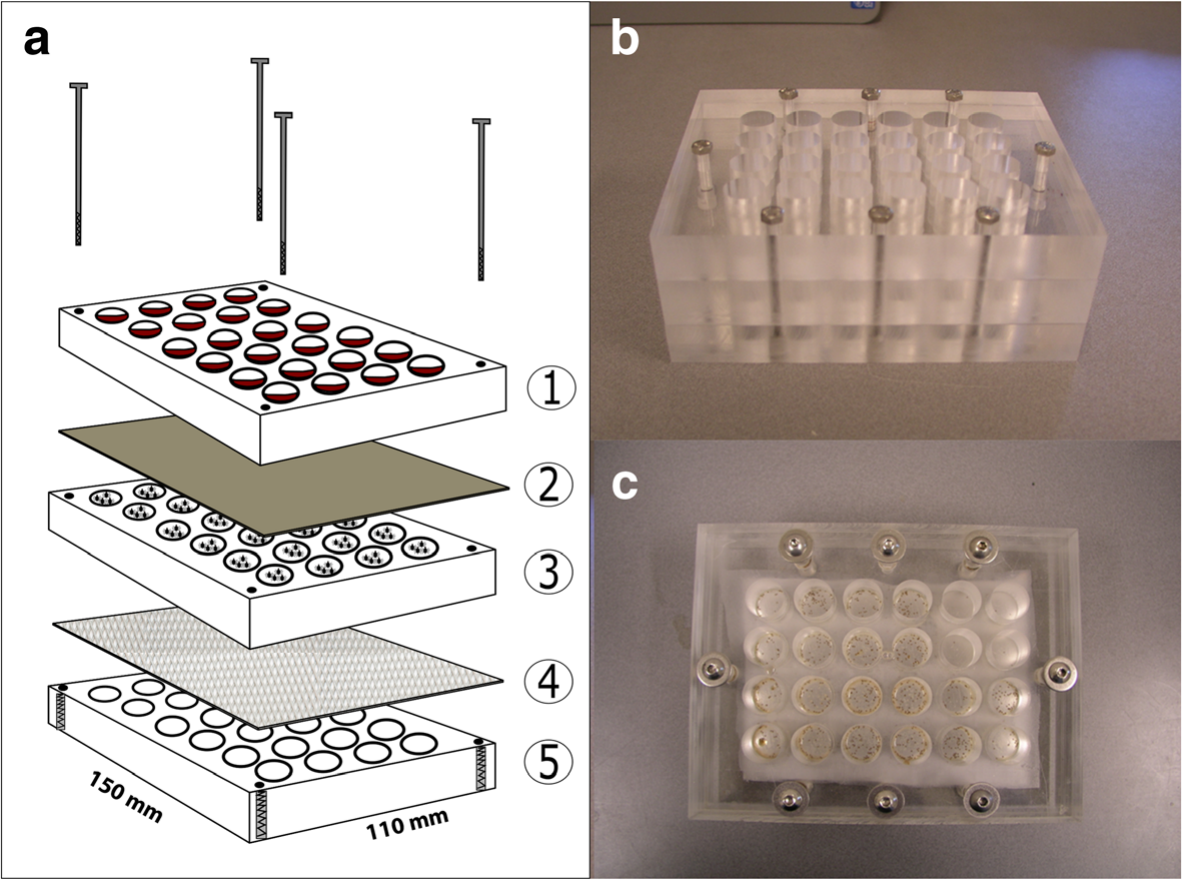
In vitro feeding unit. **a** Schematic representation: The wells of the upper layer contained the test serum or blood (1). The top was covered with an Elisa plate seal or Parafilm to maintain sterility. This layer was separated from the second layer by the feeding membrane that was placed with the silicone side facing downwards (2). The wells of the second layer contained the ticks (3). To contain the ticks, this layer was separated from the third layer with netting (4). The third layer functioned as support and allowed gas exchange between the environment and the tick compartments (5). **b** A frontal view of the unit. **c** Top view of the feeding unit, *R*. *australis* larvae can be seen through the membrane

Feeding units were designed using three 30 mm Perspex plates that were perforated to create a 24-well culture plate design (total size 150 mm length × 110 mm width, wells are 15.5 mm in diameter placed within 127 mm × 85 mm). The plates were assembled with 8 steel bolts to obtain a three-layered stack (Fig. 1). The wells of the upper layer contained the test serum or blood (Fig. 1a, layer 1). The top was covered with an Elisa plate seal or Parafilm to maintain sterility. This layer was separated from the second layer by the feeding membrane that was placed with the silicone side facing downwards (Fig. 1a, layer 2). The wells of the second layer contained the ticks (Fig. 1a, layer 3). To contain the ticks, this layer was separated from the third layer with netting (Fig. 1a, layer 4). The third layer functioned as support and allowed gas exchange between the environment and the tick compartments (Fig. 1a, layer 5).

### Feeding membrane

The basis of the feeding membranes was goldbeater’s skin of less than 30 μm thickness (Preservation Equipment Ltd, Norfolk, UK). Goldbeater’s skin is comparable to a baudruche membrane; both are made from bovine intestine. The membranes were treated with silicon to add strength and flexibility. Silicone mixture was prepared: 15 g Wacker silicone E4, 9 gr Silicone AP 200 (Sigma-Aldrich, St. louis, USA) and 5.8 g Hexane (Sigma-Aldrich). After carefully mixing, 1.5 mg silicone mixture per cm^2^ was applied with either a rubber sheet or a gloss paint roller. The siliconized membrane was left to polymerize overnight at room temperature and ambient humidity. Final membrane thickness was measured with a micrometer. Membranes with a maximal thickness of 40 μm were used for feeding.

### Feeding unit assembly

Figure 1a shows a schematic view of the feeding unit. As a first step, only the first two Perspex plates (Fig. 1a, layer 1 and 3) were assembled together with the siliconized feeding membrane (Fig. 1a, layer 2) in between, and placed upside down. Subsequently, 75 μl dichloromethane bovine hair extract was added to each well and left to dry for 30 min at room temperature to apply bovine scent to the siliconized side of the feeding membrane. Next, *R. australis* larvae were added to the wells (Fig. 1a, layer 3) (approximately 100 larvae per well). A piece of net curtain was used to cover the plate and contain the larvae, after which the lower plate was immediately mounted using the bolts (Fig. 1a, layer 4 and 5). The unit was then put upright. This system design stimulates contact between the blood (or serum) (Fig. 1a, layer 1) and the larvae; red blood cells settle on top of the feeding membrane, and larvae due to their natural anti-gravitational tendency crawl up to the underside of the membrane.

### Feeding unit operation

**Fig. 2:**
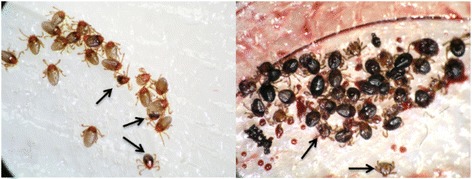
*R*. *australis* larvae on the feeding membrane. Most of the larvae have engorged with serum (*left*) or blood (*right*). Examples of larvae considered unengorged are indicated with an *arrow*

The wells of the upper plate with the baudruche side of the feeding membrane at the bottom were disinfected using 70% ethanol and left to dry. Test samples (blood or serum) were pre-warmed at 37 °C and subsequently added to the wells of the upper plate. The upper plate was sealed with an ELISA plate cover or Parafilm. The unit was placed in a dark CO_2_ incubator at 37 °C, 90% RH and 5% CO_2_ (as a feeding stimulus) for up to 72 h to allow larvae to feed. Feeding was stopped by placing the feeding unit overnight at -20 °C thus freezing the larvae. The percentage of larvae that were engorged was determined using a stereomicroscope. Larvae were scored to be engorged when having an enlarged abdomen of at least 2 times the dorsal shield (Fig. 2). To secure objectivity and prevent subconscious skewing, details about the test materials were kept secret to the evaluator (see below; Statistical evaluation).

### Bovine blood

Blood was collected from healthy tick-naive Friesian Holstein cattle in 500 ml glass bottles under vacuum with sterilized glass beads. The blood was defribinated by gently shaking. The defibrinated blood was supplemented with glucose (Sigma-Aldrich) to a concentration of 3 g/l and stored at 4 °C. Before adding the blood to the feeding well, 5 μl gentamycin (10 mg/ml; Sigma-Aldrich) and 100 μl sterile filtered ATP (0.1 M in 0.9% NaCl; Sigma-Aldrich) was added to every 10 ml of blood. Six hundred microliters of bovine blood was added to each well and changed twice daily.

### Bovine serum

To produce normal serum, blood from healthy tick-naive Holstein Friesian cattle was collected in BD Vacutainer® Plus plastic serum tubes (BD, New Jersey, USA). Blood was allowed to clot for 1 h at 37 °C, centrifuged for 15 min at 1000 RCF, serum was harvested and stored at -20 °C.

### Anti Bm86 bovine serum

Five healthy, tick-naive Friesian Holstein cattle were vaccinated twice at a 3-week interval with Bm86 produced in the *Baculovirus* expression system in water in oil adjuvant (Montanide ISA 50 V2, Seppic, Paris, France). Two weeks after the last vaccination, blood was collected for serum production. Serum was pooled before feeding.

Before serum was added to the in vitro feeding system, each 10 ml of serum was supplemented with 5 μl gentamycin (10 mg/ml; Sigma-Aldrich). Six hundred microliters of serum was added to each well and changed twice daily.

### Bm86 ELISA

Anti Bm86 bovine serum titers were determined in a sandwich ELISA. In short, rabbit anti-*Pichia* expressed Bm86 (5 μg/ml in CBB buffer) was coated overnight on a Greiner F ELISA plate at room temperature. The wells were subsequently blocked for 1 h with 200 μl/well 1% w/v BSA in 0.04 M isotonic PBS at 37 °C. Next, 100 μl/well *Baculovirus* expressed Bm86 was added to the plate (0.12 μg/ml in 1% w/v BSA in EIA-tween80 buffer) and left to incubate for 2 h at 37 °C. Serum was diluted (in 1% w/v BSA in GLD1 buffer supplemented with 10% v/v normal dog serum) and 100 μl/well subsequently added to the plate for 1 h incubation at 37 °C. Goat anti-bovine IgG-HRP (Jackson ImmunoResearch Inc., West Grove, PA, USA,) was 2500 times diluted in 1% w/v BSA in EIA-tween80 buffer and 100 μl/well added to incubate for 1 h at 37 °C. Finally, 100 μl/well substrate (185 μl TMB and 1 ml UP-buffer in 10 ml water for injection) was added and left to incubate for 15 min in the dark at room temperature. The reaction was stopped with 50 μl/well 4 N H_2_SO_4_ and OD was measured at 450 nm.

### Statistical evaluation

Samples were tested at least as quadruplicates. To prevent plate-position effects on feeding, samples were allocated to the feeding unit such that they were evenly distributed over the plate. The code was kept secret to the evaluator until after determination of the engorgement rate in each well. From the individual values the average engorgement rate was calculated. The obtained results were statistically evaluated using Graphpad Prism (Graphpad Prism 5, Graphpad software Inc.).

## Results

The goal of our study was to develop a feeding system that facilitates in vitro feeding of R. microplus in a high throughput mode. For this purpose, a 24-well format tick feeding unit consisting of Perspex plates was designed in which the feeding membrane and blood/serum are placed above the larvae, stimulating tick attachment and feeding due to the negative geotaxis of the larvae (Fig. 1). The amount of feeding medium required was 600 μl/well, which is quite low and very suitable for screening purposes.

### Membrane preparation

**Fig. 3:**
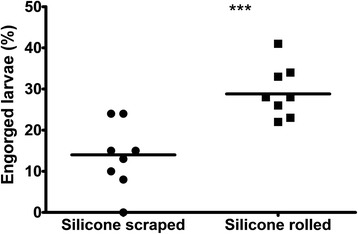
Effect of silicone application method. Percentage of ticks considered to have engorged after 48 h. Silicone application with a paint roller double the number of larvae that have engorged

Because larvae of both *R. australis* and *R. microplus* have very short mouth parts (around 70 μm) we selected goldbeater’s skin with a thickness less than 30 μm as a base. To add strength and flexibility, silicone was applied with a rubber sheet as described by Kröber & Guerin [22]. Initial results with this method revealed an engorgement rate of around 10% after 48 h of feeding on bovine blood (data not shown). Because the preparation of feeding membranes with a rubber sheet is somewhat cumbersome, we compared application of silicon by using a rubber sheet or by using a gloss paint roller. For each application method, eight wells were used. Results showed that the engorgement rate after feeding for 48 h on bovine blood was statistically significant higher using membranes prepared with the paint roller (29.4% average engorgement) as compared to membranes prepared with the rubber sheet (13.6% average engorgement; (*t*_(14)_ = 4.363, *P* = 0.0006 Fig. 3). Using the paintroller to apply the silicone mixture, it became possible to prepare membranes of 17–40 μm thickness, depending on the thickness of the used goldbeater’s skin. Microscopic examination of the membranes produced with the paint roller revealed an irregular relief.

### Optimization feeding time on bovine blood

**Fig. 4:**
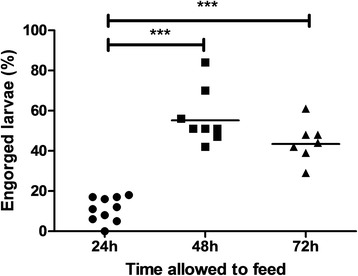
Effect of feeding time on percentage of engorged larvae. The number of engorged larvae increased significantly 48 h after feeding. Feeding for 72 h did not increase the total number of larvae that have engorged as compared to feeding for 48 h

As it was not known whether larvae that did not engorge after 48 h of feeding were incapable of feeding or would do so when given more time, we determined the engorgement rate at different time periods after initiation of feeding. Three feeding units were used to determine the engorgement rate at 24, 48 and 72 h after the start of the experiment. At each time point one feeding unit was frozen for evaluation. Results showed that the average engorgement rate of larvae that were fed bovine blood increased from 12% after 24 h to 54% after 48 h (Fig. 4). Adding another 24 h to the feeding time did not increase the average engorgement rate further.

### Feeding on bovine serum

**Fig. 5:**
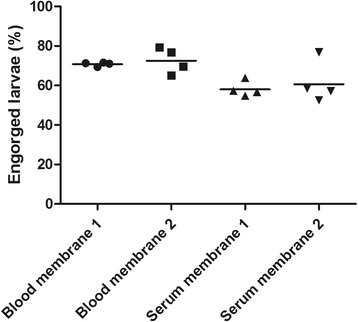
Effect of feeding medium on larval engorgement. Engorgement on defribrinated blood was compared to serum alone. In this experiment two independent feeding units were used for each feeding medium. Fifty-nine percent of the larvae with access to serum engorged compared to the 71% feeding on blood

With the basic requirements for successful feeding of larvae on blood set, we determined whether larvae would also feed on serum only. This would be of prime importance for screening tick vaccine candidates using serum of immunized calves. To also test variation due to different batches of siliconized membranes, two different units were set up with membranes from different batches. In each of the units, part of the wells was filled with bovine blood and another part with bovine serum. The larvae were allowed to feed for 48 h on these substrates after which the engorgement rate was determined. Results showed that the engorgement rate of larvae feeding on bovine serum was somewhat lower (59%) than that observed when larvae were fed on bovine blood (71%; Fig. 5). This difference was statistically significant (*t*_(14)_ = 3.858, *P* = 0.0017). There was no apparent difference between the engorgement rates obtained with the two different batches of siliconized membranes.

### Effect of anti Bm86 bovine serum

**Fig. 6:**
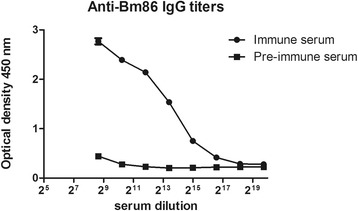
Specificity and IgG response against Bm86. Serum reactivity was tested with ELISA. *Baculovirus* expressed Bm86 was captured with Rabbit IgG raised against *Pichia* expressed Bm86. The 2log end titer of immune serum is 17

**Fig. 7:**
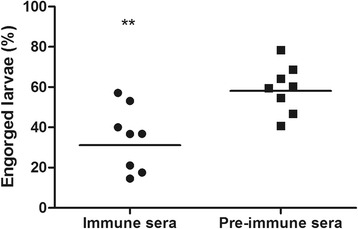
Effect of Bm86 vaccination on percentage of larvae that have engorged. Vaccination reduced the number of engorged larvae by 42%

To validate the use of the feeding system for the selection of putative tick vaccine candidates we used Bm86 midgut antigen as an example. Serum was prepared from calves prior to and after immunization with recombinant Bm86 produced in the Baculovirus expression system. An ELISA capturing recombinant *Baculovirus* expressed Bm86 with rabbit anti-Bm86 IgG against *Pichia* expressed Bm86 was used to measure the antibody titer of the anti-Bm86 calf serum. Results showed that vaccination with Bm86 resulted in a 2log antibody end titer of 17 (cut off calculation: Bmin*2) (Fig. 6). Next, both sera were used to feed larvae in vitro to determine the effect of anti Bm86 antibodies on engorgement. Each serum was tested in eight-fold, larvae were allowed to feed for 48 h. Results showed that the engorgement rate of larvae that were fed on anti-Bm86 serum was reduced by 42% as compared to controls (Fig. 7). This result was statistically significant (*t*_(14)_ = 3.497, *P* = 0.0036).

## Discussion

The search for effective anti-tick vaccines is currently restricted by the use of calves to determine the efficacy of immunization in vaccination-challenge experiments. An in vitro tick feeding system that allows determining the effect of sera from vaccinated calves on tick viability would be advantageous. In vitro feeding systems using artificial feeding membranes have been developed for several different tick species, but until now no such system has been developed for *R. microplus* larvae. This has been hypothesized to be due to the fact that *R. microplus* have a very short hypostome (approximately 70 μm) [23, 31, 32], which is too short to penetrate the type of artificial membranes that are used for feeding of ticks in vitro [23]. Here we show that a feeding membrane based on goldbeater’s skin that is coated with silicon effectively supports feeding of *R. australis* (formerly *R. microplus*) larvae in vitro. *R. australis* ticks were considered to be *R. microplus* until comprehensive morphological and genetic analysis showed that this group of ticks should be divided into two species [31]. However, since the larvae from both species have the same hypostome length and both species are affected by Bm86 vaccination, the results presented here with *R. australis* can be easily translated to *R. microplus* larvae [31]. In the current in vitro assay, the method used to coat the goldbeater’s skin with silicon appeared to be an important variable; application of silicon with a gloss paint roller produced better membranes as compared to application by wiping with a rubber sheet. It is suggested here that the irregular surface texture produced with the paint roller is crucial, probably because it provides support for the larvae to attach to the membrane and the fact that the membrane is at some points thinner than at other points allows successful penetration by the hypostome. Importantly, between membranes there was little variation in larval feeding, illustrating the reproducibility of the membrane production. The engorgement rate of the larvae was determined by estimating their size visually, using a stereomicroscope. It could be argued that measuring larval weight after feeding, instead of size, would give a more objective measurement of engorgement. Unfortunately, the attachment to the membrane at the moment of scoring and the number of larvae used (approx. 2400 in one in vitro feeding unit) make it technically nearly impossible to remove all larvae undamaged for accurate weighing.

The results presented here show that there is considerable variation in the engorgement rate per well. Because of this variation it is important to have at least quadruplicate measurements to calculate reliable average engorgement rates. The 24-well plate set-up presented here, enables testing different feeding substrates in a single feeding unit. Additional advantages of the system are that the feeding substrates can be presented to the ticks aseptically, relatively small volumes of blood/serum are required, the feeding membrane is located at the top of the wells that contain the larvae (larvae tend to crawl upward), and relatively large numbers of larvae can be used per well, which adds to the accuracy of measuring engorgement rates.

Results showed that larvae achieved the maximum engorgement rate after 48 h of feeding; adding another 24 h did not increase the engorgement rate further. Hence, assessing the inhibitory effect of anti-tick compounds (be it acaricides or sera from immunized animals) can be completed in two days in vitro if the targeted antigen is expressed in the first 48 h after tick attachment. For antigens expressed at later time points longer feeding time might be required. This is however a major advantage over in vivo vaccination-challenge experiments in calves that are currently used to determine the effect of vaccination on tick infestation. Such experiments take on average 3 to 4 weeks before larvae have developed to fully engorged female ticks. Clearly, in vivo challenge with larvae does not only estimate the effect of treatment on larvae but also on nymphs and adult ticks, because *R. microplus* and *R. australis* are one-host ticks with most of the larvae developing to fully engorged females on the same host. The system presented here is validated to test the effects of anti-tick compounds on larvae. Although the system enables the development of larvae to nymphs (up to 11% of applied larvae moulted into nymphs after two-week incubation in a CO_2_ incubator at 37 °C, 90% RH and 5% CO_2_ with only an initial 600 μl tick naive bovine serum added to the feeding unit; data not shown), it remains to be investigated whether it can also be used to feed nymphs. It is not likely that it can be used to feed adults because these are too large for a 24-well format. Alternatively, a 6-well format could be developed for mature stages.

We observed variation in the engorgement rate among different experiments using the same substrates for feeding. Given the fact that the membranes could be prepared in a reproducible manner, the most likely explanation for this variation is due to differences in the viability of the larvae that were used. Consequently, it is advised to test the effect of anti-tick compounds in the feeding substrate using larvae of similar age derived from a single batch.

Although it has been shown before that larvae feed on tissue fluids containing very few erythrocytes [33, 34], it remained to be determined whether serum alone would suffice, especially if the system is to be used to determine the effect of anti-tick antibodies on larval development. Kemp et al. have shown in 1975 that *R. microplus* larvae indeed feed on medium enriched with bovine serum proteins [28]. In addition, it has been shown that serum as a blood meal does not repel larval attachment, however it is not clear if the larvae feed on serum alone [27]. Using the in vitro system presented here, larvae could feed on serum alone, although engorgement rates were somewhat reduced (59% fed on serum as compared to 71% on defibrinated blood). Hence, the system appears suited to aid in the selection of tick vaccine candidate antigens by feeding serum from immunized animals and comparing the engorgement rates with that obtained with normal serum controls.

For the present investigation, Bm86 was chosen as a reference antigen. Pooled serum from calves that were vaccinated with recombinant Bm86 reduced engorgement of larvae by 42%. This indicates that the Bm86 antigen is indeed expressed in the larval stage of *R. microplus* as has been found previously by qPCR [35], and more importantly is exposed to ingested antibodies directed against the antigen. Since *R. microplus* is a one-host tick, one would expect that antibodies against Bm86 would also affect nymphs next to larvae and adults. The reduction in engorgement by Bm86 immune sera in vitro is difficult to compare to earlier published in vivo results. Aside from differences in antigens, adjuvants and vaccine formulations used to vaccinate the cattle, field circumstances such as cattle breed, presence of different tick strains and epidemiological situations all influence efficacy of vaccination.

## Conclusion

A 24 well in vitro feeding system has been developed to successfully feed *Rhipicephalus australis* larvae in vitro. By applying a silicone mixture with a paint roller on a baudruche sheet, membranes < 40 μm thick were made that can facilitate the short hypostome of *R. australis* and *R. microplus* larvae. Using these membranes in the 24-well in vitro system, larvae were able and willing to feed on defibrinated blood as well as serum alone. Feeding larvae on sera raised against the reference antigen Bm86 reduced tick engorgement with 42%, thereby confirming the feeding systems ability to discriminate anti-tick effects in vitro.

Importantly, it is envisaged that the feeding system can be very useful to determine the potential protective effect of vaccines that contain a mixture of one or more tick antigens. Instead of vaccinating cattle with a plethora of different vaccine formulations, the system allows testing the effect of combinations of anti-tick antibodies by mixing different mono-specific antisera directed against individual tick antigens.

In summary, the in vitro feeding system presented here allows reproducible feeding of *R. australis* larvae on bovine blood and serum. The system can be used for screening tick vaccine candidate antigens and as such accelerate the development of improved anti-tick vaccines.
